# Emergence and Diversification of Dengue 2 Cosmopolitan Genotype in Pakistan, 2011

**DOI:** 10.1371/journal.pone.0056391

**Published:** 2013-03-08

**Authors:** Mohammad A. Khan, Esther M. Ellis, Hasitha A. Tissera, Mohammad Y. Alvi, Fatima F. Rahman, Faisal Masud, Angelia Chow, Shiqin Howe, Vijaykrishna Dhanasekaran, Brett R. Ellis, Duane J. Gubler

**Affiliations:** 1 Services Institute of Medical Sciences, Services Hospital, Lahore, Pakistan; 2 Program in Emerging Infectious Diseases, Duke-NUS Graduate Medical School, Singapore, Singapore; 3 Epidemiology Unit, Ministry of Health, Colombo, Sri Lanka; University of Texas at El Paso, United States of America

## Abstract

Major dengue epidemics have been observed in the Indian subcontinent since the 1980s and have occurred with increased hospitalizations and mortality. In 2011, the first major epidemic of dengue occurred in Lahore, the second largest city in Pakistan, and resulted in 21,685 confirmed cases and 350 deaths. To investigate the possible viral causes for the increased epidemic activity, we determined the predominant serotype and characterized the viruses genetically. Of 50 patients carefully selected as probable dengue fever or dengue hemorrhagic fever, 34 were positive by virologic testing (i.e. PCR and/or virus isolation). DENV-2 was detected in 32 patients and DENV-1 in two. A total of 24 partial and three full DENV genomes were sequenced. Phylogenetic analyses of the capsid (C), pre-membrane (prM), and envelope genes comprising 2500 nucleotides in length indicated that all DENV-2 isolates in Pakistan since 2007 form a monophyletic lineage that is endemic in the country. These viruses were all of the cosmopolitan genotype (IV) and most closely related to viruses isolated in India and Sri Lanka in the past two decades. Phylogenetic analyses of data currently available in GenBank suggest that the Cosmopolitan genotype has diverged into two geographically distinct sub-lineages: sub-lineage IV-a has only been observed in Southeast Asia, China and Oceania, while IV-b is prevalent in the Indian subcontinent. These results highlight the increased diversity of dengue viruses as they spread geographically within the region.

## Introduction

Dengue fever (DF) has been reported in the Indian subcontinent for many decades, but major epidemics were not reported until the late 1980s [1]. Sero-epidemiological evidence, from the 1960s and 1970s, demonstrated high flavivirus antibody prevalence rates in Pakistan, but the specific viruses present and extent of dengue transmission were uncertain [2]. The first documented dengue epidemic in Pakistan did not occur until 1994, which included the first reported cases of dengue hemorrhagic fever (DHF) [3]. Sporadic cases of DHF associated with small dengue outbreaks occurred over the next 10 years, but few viruses were characterized [4]. In 2005, a large epidemic of DENV-3 occurred in Karachi [4]. Since then, Pakistan has experienced major or minor outbreaks of DF/DHF annually, caused mainly by DENV-2 and DENV-3, although DENV-1 and DENV-4 have been isolated in recent years [5], [6], [7]. In 2011, Lahore, capital of the Punjab, experienced its first major dengue epidemic, with 21,685 confirmed cases and 350 deaths (Directorate General Health Services, Lahore). Here we describe clinical and virologic aspects of a small subset of carefully selected patients admitted to one hospital.

## Materials and Methods

### Ethics Statement

The study was approved by the ethical committee of Services Institute of Medical Sciences/Services Hospital, Lahore, Pakistan. All patients provided oral informed consent to participate in the study. The reason for not obtaining written consent was that this study was carried out in the midst of a large epidemic when there was an atmosphere of fear among the patients, did not want to spend time in reading and signing the consent in ER. In some cases patients were illiterate (unable to read and write or sign).

The attending doctor in ER along with the doctor obtaining the blood sample explained the need for this study to the patients and obtained verbal consent from the patients directly, and in case of minors from the parents and recorded them in the performa. The ethical committe of Services Hospital, Lahore, did grant permission for verbal consent."

### Study Population

Fifty patients of various ages (median age = 22) (Table 1) presenting with high suspicion of DF or DHF at Services Hospital, Lahore, were enrolled in this study. All patients fulfilled a majority of the following criteria: 1) high grade fever with chills and rigors without any focus; 2) severe headache and/or retro orbital pain; 3) generalized body aches and pains; 4) generalized body rash; and 5) thrombocytopenia and/or leukopenia. Patients with fever and any other evident focus of infection were excluded. Blood samples were taken during the first 5 days of illness and serum was stored at −70°C until the samples were sent on dry ice via courier to Duke-NUS Graduate Medical School in Singapore for serology, virus isolation and sequencing.

**Table 1:** Serologic and virologic confirmation of dengue virus infection in Pakistan patients by age group.

Age-Group	No.	Male	Female	IgM^1^ (%)	IgG^1^ (%)	Isolation^2^	PCR	Current/recent infection Total^3^
5–9	2	0	2	2/2 (100)	2/2 (100)	0/2 (0)	0/2 (0)	2/2 (100)
10–14	7	6	1	6/7 (86)	6/7 (86)	2/7 (29)	4/7 (57)	7/7 (100)
15–19	5	5	0	3/5 (60)	4/5 (80)	3/5 (60)	3/5 (60)	4/5 (80)
20–29	17	11	6	10/17 (59)	11/17 (65)	13/17 (76)	13/17 (76)	17/17 (100)
30–39	11	6	5	6/11 (55)	7/11 (64)	7/11 (64)	7/11 (64)	10/11 (91)
40–49	3	1	2	0/3 (0)	1/3 (33)	3/3 (100)	3/3 (100)	3/3 (100)
50+	5	5	0	3/5 (60)	5/5 (100)	2/5 (40)	3/5 (60)	5/5 (100)
Total	50	34	16	30/50 (60)	36/50 (72)	30/50 (60)	33/50 (66)	48/50 (96)

1 IgM and IgG specific dengue antibody detected using an in-house Luminex platform based microsphere-bead immunoassay. ^2^Virus isolation was done by intra-thoracic circulation of mosquitoes. ^3^Combined IgM, PCR and virus isolation results

### Serological Testing

Specific anti-dengue IgM and IgG antibody was detected using an in-house Luminex platform based microsphere-bead immunoassay (unpublished). In short, DENV-2 whole virus antigen was coated onto microsphere beads to detect DENV-1-4 specific antibodies and raw data were transformed to give median fluorescence intensity (MFI) for anti-dengue IgM and IgG.

### Virus Isolation and PCR

Virus isolation was performed in C6/36 *Ae. albopictus* cells and by intra-thoracic inoculation of *Ae. aegypti* mosquitoes, as previously described [8], [9]. RNA was extracted from sera and tested by a serotype specific quantitative reverse transcription PCR qRT-PCR [10]. Briefly, reaction mixtures for detection and quantitation of DENV-1 and DENV-2, 5 µL of RNA were combined with 50 pmol of each primer and 9 pmol of probe in a 50 µL reaction using the SuperScript III Platinum One-Step quantitative RT-PCR system (Invitrogen-ABI). Each reaction mixture contained a single DENV serotype primer pair and probe; therefore, four separate singleplex assays were carried out for each RNA sample.

### Sequencing and Analysis

Capsid, pre-membrane, and envelope genes were sequenced (∼2.5 kb) directly from serum PCR products as previously described [11]. Two DENV-1, confirmed by virus isolation, were also sequenced directly from patient serum. Full genome sequences were generated for 3 representative DENV-2, also directly from patient serum samples. The nucleotide sequences reported in this paper have been deposited in the GenBank database (accession numbers JX042490- JX042514). Sequence assembly, editing and multiple sequence alignments were performed using Geneious (Biomatters) and Se-Al (Se-Al - http://tree.bio.ed.ac.uk/software/) software. Maximum likelihood trees using the GTR+I+G nucleotide substitution model were generated using RAxML [12].

## Results

All age groups studied had similarly high infection rates (Table 1). Over half (28/50; 56%) of the patients selected were young adults between the ages of 20 to 40, and the majority (86%) were males. Overall, 48/50 (96%) of patients were confirmed as a current or recent dengue infection. Virus was isolated from 30 of 50 patients (60%) and 33 (66%) were positive by PCR. Four patients positive by PCR were negative by virus isolation and one patient positive by virus isolation was negative by PCR. The virus negative, IgM positive patients were most likely current infections, given the clinical presentation, although without the availability of paired sera samples this remains uncertain.

All patients were febrile and had presented early to the hospital. The average duration of fever on admission was 3.6 days. Myalgias (90%) and headache (84%) were the most common symptoms. All patients had leukopenia and thrombocytopenia. Frank hemorrhagic manifestations were rare, but 3/50 (6%) had DHF confirmed by plasma leakage and two others had narrowing of pulse pressure to less than 20 mm Hg. A high proportion of patients (72%) had IgG antibody in their acute blood sample, suggesting prior infection with dengue or another flavivirus. Although the sample set studied is small, the IgM age specific prevalence supports the conclusion that all age groups were highly susceptible to DENV-2 infection.

Of 34 patients with virologic confirmation (i.e. PCR and/or virus isolation), 32 (94%) were DENV-2 positive and two were DENV-1 positive. The high virus isolation rate prompted us to measure viremia levels in all 33 PCR positive patients, which averaged 6.8 log10 genomic equivalents (GE) per mL for DENV-2 (range 3.5 to 8.0 log10 GE per mL). Average titers were higher for DENV-1 patients (i.e. 8.9 log10 GE), but the small number of DENV-1 samples was not sufficient to draw specific conclusions. There were no significant differences in virus titers according to date of presentation.

**Figure 1:**
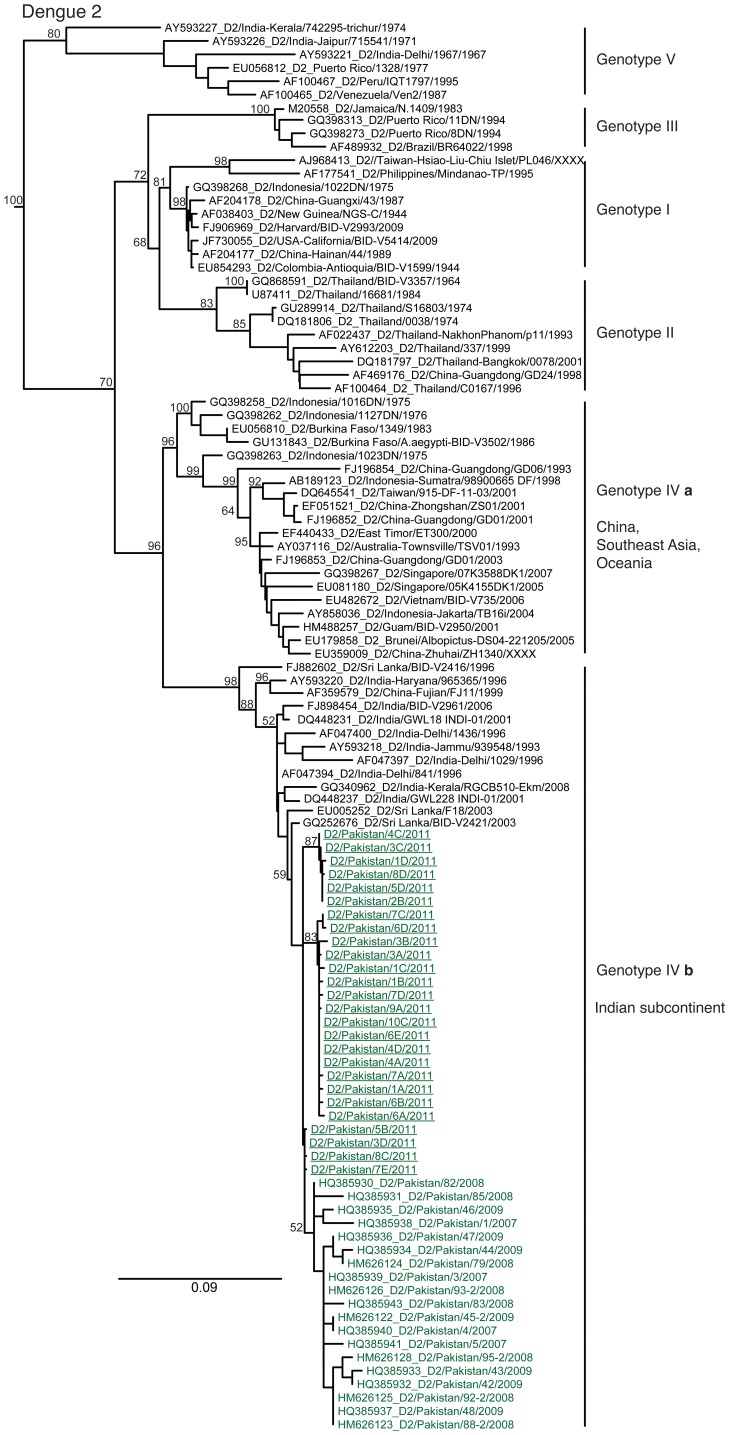
Phylogenetic relationships of Pakistan DENV-2. Phylogenetic relationships of Dengue viruses type 2 isolated from severe cases in 2011 in Lahore, Pakistan. Numbers at branch nodes indicate maximum likelihood bootstrap values. Underlined virus names represent newly generated sequences. Analyses were based on 2373 nts of the capsid, pre-membrane, and envelope gene region. Trees are midpoint rooted. Scale bars indicate nucleotide substitutions per site.

Phylogenetic analyses of the 2011 viruses showed the Pakistan DENV-2 lineage to be largely unchanged since 2007 (Figure 1; Cosmopolitan genotype IVb). These viruses were all most closely related to viruses isolated in India and Sri Lanka in the past two decades, and cluster separately from other DENV-2 Cosmopolitan genotype viruses (Figure 1; Cosmopolitan genotype IVa). Full genome sequences generated for 3 representative 2011 DENV-2 isolates showed only 2 unique amino acid changes from earlier genotype IVb isolates (I1307T in the NS2A and S3230N in the NS-5) from Pakistan.

**Figure 2:**
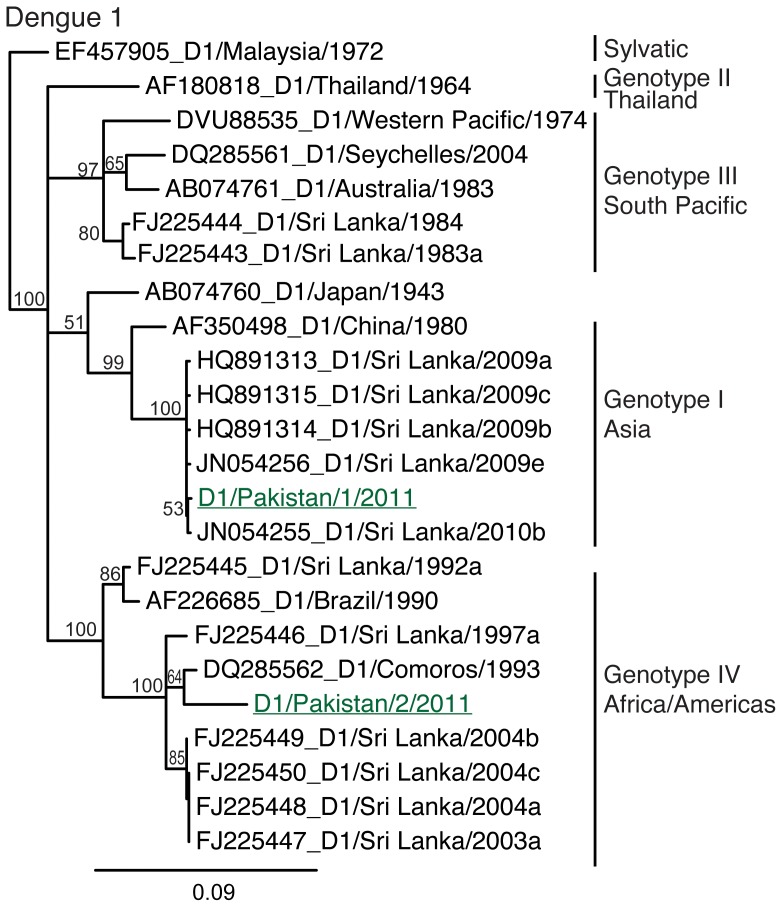
Phylogenetic relationships of Pakistan DENV-1. Phylogenetic relationships of Dengue viruses type 1 isolated from severe cases in 2011 in Lahore, Pakistan. Numbers at branch nodes indicate maximum likelihood bootstrap values. Underlined virus names represent newly generated sequences. Analyses were based on 505 nts of the envelope gene region. Trees are midpoint rooted. Scale bars indicate nucleotide substitutions per site.

The DENV-1 isolates (Figure 2) clustered in two different DENV-1 genotypes (I and IV). One of the DENV-1 viruses (D1/Pakistan/1/2011) was closely related to DENV-1 (Genotype I, Asia) (Figure 2), which was responsible for a large DHF epidemic in Sri Lanka in 2009–2010 [13]. The second isolate (D1/Pakistan/2/2011) was more closely related to a DENV-1 that has been endemic in Sri Lanka for many years.

## Discussion

DENV-2 appears to have been the predominant serotype circulating in Pakistan since 1994, although DENV-3 caused epidemics in 2005 to 2008 [5]. The predominance of DENV-2 is not unexpected as this is a prevalent serotype worldwide and has been associated with epidemics of severe disease [13]. The overall phylogeny of DENV-2 sequences included here highlights the increased diversity of dengue viruses as they spread geographically within the region. Our results also show that genotype IV has diverged into two geographically distinct sub-lineages in the past two decades: sub-lineage IVa, which has only been observed in Southeast Asia, China and Oceania, while IVb has only been reported in the Indian subcontinent (Fig 1).

Detection of DENV-1 during the most recent outbreak is also not entirely unexpected based upon the hyper-endemicity of dengue in the Indian subcontinent [14]. DENV-1 has been detected in Pakistan since at least the 1990's and was the predominant serotype circulating in neighboring India from 1997–2004, until it was replaced by DENV-2 [5]. The presence of two DENV-1 genotypes is interesting and similar viruses to these had previously been reported in Sri Lanka in 2004 and 2009. The genotypes identified are interesting because they have previously been associated with contrasting clinical severities and clade replacement in Sri Lanka [15]. However, the few sequences currently available from Pakistan cannot be used to reliably discern the patterns to which multiple DENV-1 genotypes may be maintained endemically or periodically introduced from elsewhere.

The overall evolution of epidemic DHF in Pakistan has followed a pattern frequently observed in other dengue endemic countries, beginning with periodic dengue epidemics and sporadic DHF cases, followed by the development of hyper-endemicity (i.e. the co-circulation of multiple virus serotypes), which ultimately culminates in major epidemics of severe disease. The magnitude and the severity of the 2011 Lahore epidemic could suggest the introduction of a novel strain or subtype of DENV-2; however, the phylogenetic analysis presented here indicates a locally evolved strain as the cause. Although it is still not entirely clear what drove this particularly severe epidemic, viral genetic changes, the circulation of multiple serotypes, genetic and immunological factors, ecological perturbations, and entomological factors may have contributed. Accumulating evidence suggests that even small genetic changes may lead to virus subtypes or lineages that have greater fitness, greater epidemic potential and perhaps greater virulence [16]–[20]. This should be of significant concern and similar genetic changes in the non-structural genes of local strains of DENV-2 (South Pacific), DENV-3 (Sri Lanka) and DENV-4 (Puerto Rico) have previously been associated with changes in DENV epidemic potential [17]-[20]. Full genome sequences from additional Pakistan isolates and neighboring regions are needed to ultimately determine if a sub-lineage with increased epidemic potential may have evolved locally to cause the Lahore epidemic.
